# Increasing Antimicrobial Resistance to First-Line Therapies in Chronic Endometritis: A 2020–2024 Cross-Sectional Study

**DOI:** 10.3390/jcm14144873

**Published:** 2025-07-09

**Authors:** Ettore Cicinelli, Francesco Di Gennaro, Antonia Gesario, Daniela Iachetti Amati, Giacomo Guido, Luisa Frallonardo, Annalisa Saracino, Antonella Vimercati, Rossana Cicinelli, Pierpaolo Nicolì, Amerigo Vitagliano

**Affiliations:** 1 Unit of Obstetrics and Gynecology, Department of Interdisciplinary Medicine (DIM), University of Bari Aldo Moro, Policlinico of Bari, Piazza Giulio Cesare 11, 70124 Bari, Italy; 2 Clinic of Infectious Diseases, Department of Precision and Regenerative Medicine and Ionian Area (DiMePRe-J), University of Bari Aldo Moro, Policlinico of Bari, Piazza Giulio Cesare 11, 70124 Bari, Italy; 3 Laboratorio Analisi F. Ditonno SRL, 70122 Bari, Italy; 4 Department of Obstetrics, Gynecology, and Reproductive Medicine, Hospital Foch, the University of Paris Ouest (UVSQ), 78000 Paris, France

**Keywords:** chronic endometritis, treatment, microbial culture, antimicrobial resistance, hysteroscopy

## Abstract

**Objectives:** In the context of the global rise in antimicrobial resistance (AMR), this study aimed to investigate temporal trends in AMR among pathogens isolated from endometrial cultures of patients diagnosed with chronic endometritis (CE). **Methods:** This cross-sectional study included 244 women consecutively diagnosed with CE at the Gynecology Unit of the University of Bari, Italy, between January 2020 and June 2024. Exclusion criteria were (i) previous CE diagnosis or treatment; (ii) antimicrobial use in the month prior to hysteroscopy and biopsy; (iii) use of oral or vaginal prebiotics/probiotics or contraceptives in the three months prior; (iv) known immunosuppression; and (v) hypersensitivity to quinolones or macrolides. CE was diagnosed using hysteroscopy combined with endometrial histology and microbial culture. Specifically, in cases with hysteroscopic signs suggestive of CE, endometrial biopsies were obtained using a Novak curette and processed for histological and immunohistochemical analyses, as well as for microbial identification and antimicrobial susceptibility testing in accordance with EUCAST guidelines. The primary outcomes were the prevalence of CE-associated pathogens and their AMR profiles. **Results:** The median age at CE diagnosis was 33 years (range 26–44). The most frequently isolated pathogens were *Enterococcus faecalis* (26.2%), *Escherichia coli* (19.3%), and *Ureaplasma urealyticum* (16.4%). High AMR rates were observed, with increasing trends over time. Ampicillin resistance reached 98.5% (63/64), penicillin resistance 30.8% (16/52), and extended-spectrum beta-lactamase (ESBL) positivity 34.7% (25/72), all with statistically significant trends (*p* < 0.001). Resistance to commonly used first-line antimicrobials, such as tetracyclines, quinolones, and nitroimidazoles, was also substantial. **Conclusions**: This study highlights a significant increase in AMRs among microorganisms responsible for CE between 2020 and 2024. As a result, empirical first-line antimicrobial therapies commonly used to treat patients with CE may be increasingly ineffective in a growing number of cases. This underscores the need for improved and targeted diagnostic and therapeutic strategies to effectively manage CE and prevent treatment failures. Strengthening surveillance systems, implementing antimicrobial stewardship programs, and enhancing patient education may help counter the growing threat of AMR.

## 1. Introduction

Chronic endometritis (CE) is an infection-related inflammatory condition of the endometrium, most commonly caused by intestinal bacteria such as *Escherichia coli*, *Klebsiella* spp., and *Enterococcus faecalis*, as well as other microorganisms including *Mycoplasma* spp. and *Candida* spp. [1,2,3]. Risk factors include constipation, recurrent urinary tract infections, vaginitis, and certain lifestyle-related factors such as stress.

CE has growing clinical and public health relevance due to its frequent association with unexplained infertility, recurrent pregnancy loss (RPL), and repeated in vitro fertilization (IVF) failure [4]. The estimated prevalence in women with RPL or IVF failure is as high as 60% and 66%, respectively [4,5]. Furthermore, very recent evidence [6] has highlighted a high prevalence of CE in women with non-structural abnormal uterine bleeding (AUB), which represents a major indication for gynecologic consultation worldwide. This finding, together with the significant improvement in bleeding patterns observed following CE resolution compared to cases with persistent inflammation [6], supports the hypothesis of a possible causal relationship between the two conditions, warranting further research on this topic.

The clinical presentation of CE is often subtle, and the condition is frequently asymptomatic or associated with mild, nonspecific symptoms such as spotting, pelvic discomfort, or leucorrhea. This makes diagnosis difficult and complicates efforts to estimate its true prevalence in the general population.

Currently, no universally accepted diagnostic criteria exist due to limitations across all available methods. The most validated approach is histological evaluation of endometrial tissue combined with immunohistochemistry for plasma cell markers such as CD138 or MUM1 [7,8,9]. However, the focal nature of endometrial biopsy and the uneven distribution of plasma cells within the endometrial stroma can lead to false-negative results. Hysteroscopy can help identify visual signs suggestive of CE [10,11], but its diagnostic accuracy depends largely on the operator’s experience. A third option is microbial culture of the endometrium [1], which may identify specific pathogens and guide targeted antimicrobial therapy. This method, however, is time-consuming and limited by the inability to detect non-culturable organisms.

Over this background, in clinical practice, empirical antimicrobial treatment with quinolones, nitroimidazoles, lincosamides, or tetracyclines is often used, especially in patients seeking pregnancy. Antimicrobial therapy has proven effective in resolving CE and improving reproductive outcomes [5,12,13]. However, successful treatment may be compromised by the increasing threat of antimicrobial resistance (AMR) [14,15]. AMR is responsible for approximately 4.95 million deaths each year and has been recognized by the World Health Organization as one of the top ten global threats to public health (https://www.ecdc.europa.eu/en/news-events/whoecdc-report-antimicrobial-resistance-threatens-patient-safety-european-region, accessed on 20 May 2025). It reduces the effectiveness of antimicrobial agents, making infections more difficult to treat [16], especially when antimicrobials are used empirically without prior microbial culture or susceptibility testing. This issue is further complicated in CE by the frequent use of antimicrobials to manage coexisting infections, such as recurrent cystitis.

Despite the growing relevance of AMR, data on resistance patterns among microorganisms associated with CE remain limited. The aim of this study was to evaluate the prevalence of microorganisms isolated from endometrial cultures in patients with CE and to assess their AMR profiles.

## 2. Materials and Methods

Patients suffering from CE were admitted between January 2020 and June 2024 at the Gynecology Unit of the Policlinico of Bari, Italy, and were consecutively enrolled in this cross-sectional study.

The exclusion criteria were (i) previous diagnosis and treatment for CE; (ii) antimicrobial use during the month preceding hysteroscopy and biopsy; (iii) use of oral or vaginal prebiotics/probiotics, as well as contraceptives, during the three months preceding hysteroscopy and biopsy; (iv) known immunosuppression; and (v) known hypersensitivity to quinolones or macrolides.

Patients were considered positive for CE when both hysteroscopic and histological/immunohistochemical (HIS/IHC) criteria were simultaneously met. Fluid hysteroscopy was performed between days 6 and 12 of the menstrual cycle, following previously published diagnostic criteria for hysteroscopic identification of CE [11]. After the end of the menstrual cycle following hysteroscopy, patients with hysteroscopic signs of CE underwent endometrial biopsy for HIS/IHC evaluation and microbial culture. Endometrial sampling was performed using a 3 mm Novak cannula, which was rotated during aspiration to collect the largest possible specimen from multiple areas of the uterine cavity, as previously described [1,17]. During the biopsy procedure, particular care was taken to avoid vaginal contamination. Endometrial samples were divided into two aliquots: one was sent for HIS/IHC analyses and the other for microbial culture and antimicrobial susceptibility testing. For this study, we recorded culture results and AMR patterns only in patients who tested positive upon HIS/IHC examination.

All resistance and susceptibility tests were conducted according to the European Committee on Antimicrobial Susceptibility Testing (EUCAST), Version 14.0, effective from 1 January 2024 (http://www.eucast.org, accessed on 20 May 2025). *Enterococcus* species were tested for ampicillin susceptibility. *Escherichia coli* and *Klebsiella* species were classified as extended-spectrum beta-lactamase (ESBL) producers if resistant to both penicillins and cephalosporins. *Candida* species (e.g., *Candida krusei*, *Candida albicans*) were tested for fluconazole resistance. *Streptococcus agalactiae* and *Streptococcus bovis* were evaluated for penicillin resistance. *Ureaplasma urealyticum* was classified as monodrug-resistant (MONO-R) if resistant to macrolides, fluoroquinolones, or tetracyclines and as multidrug-resistant (MDR) if resistant to two out of these three antimicrobial classes. Additionally, for each isolated microorganism, resistance to commonly used empirical first-line treatments was assessed, including tetracyclines, quinolones, lincosamides, and macrolides. Although metronidazole mainly targets anaerobic bacteria, its resistance profile was also analyzed for descriptive purposes, reflecting its occasional empirical use in pelvic infections in clinical practice, despite the limited involvement of strict anaerobes in CE.

EUCAST classifies susceptibility into three categories:

- S—Susceptible to standard dosing regimen, meaning there is a high likelihood of therapeutic success with standard dosing.
- I—Susceptible to increased exposure, meaning there is a high likelihood of therapeutic success with adjusted dosing or higher local concentration.
- R—Resistant: high likelihood of therapeutic failure.

The study was approved by the local Ethics Committee (ID 2456, November 2022).

### Statistical Analysis

The characteristics of the analyzed samples were described using percentage distributions presented in contingency tables. For age at diagnosis, the mean and standard deviation were calculated. To assess trends within the contingency tables, *p*-values were computed using the test for ordered proportions. The effect of age, treated as a continuous variable, on resistance outcomes was evaluated using binomial logistic regression models. To analyze resistance trends over time, a nested binomial logistic regression model was applied. The dependent variable was resistance outcome, categorized as pan-sensitive (Pan-S), MONO-R, or MDR, while the year of diagnosis and age were included as independent variables. In this model, temporal nesting was used, with resistance outcomes grouped by year of diagnosis to account for within-year clustering and to assess temporal trends.

All analyses were performed using RStudio IDE (version 2023.12.0.369) in the R statistical computing environment (version 4.2.2). A *p*-value of less than 0.05 was considered statistically significant.

## 3. Results

A total of 244 patients with a new diagnosis of CE were enrolled during the recruitment period. The median age at diagnosis was 33 years (range: 26 to 44).

The distribution of isolated pathogens varied over the years from 2020 to 2024 (Table 1). The most prevalent species identified was *Enterococcus faecalis*, which accounted for 26.2% of infections (64 cases). This pathogen showed a relatively stable presence across years, with the highest occurrence in 2022 (32.6%) and the lowest in 2020 (16.7%).

*Escherichia coli* was the second most common pathogen, detected in 19.3% of patients (47 cases). Its prevalence peaked in 2024 (30.4% of isolates for that year) and was lowest in 2020 (14.3%).

*Klebsiella pneumoniae* was identified in 9.8% of cases (24 patients), with a peak incidence in 2023 (18.4%).

*Ureaplasma urealyticum* was isolated in 16.4% of patients (40 cases), with higher detection rates in 2020 (28.6%) and 2021 (18.9%).

*Candida* species were less frequently isolated. *Candida albicans* was identified in 1.2% of patients (three cases), with isolated occurrences in 2020, 2021, and 2022. Other *Candida* species, including *Candida glabrata*, *Candida krusei*, and *Candida parapsilosis*, were rare, each accounting for less than 1% of total isolates.

Ampicillin resistance was detected in 64 out of 65 patients, with only 1 sensitive case observed in 2021. The yearly distribution (Table 2) showed consistently high resistance levels throughout the entire study period.

Among 72 patients tested for ESBL production, a significant upward trend was observed (Table 3). While no ESBL-positive cases were detected in 2020, the proportion of positive isolates increased sharply in the following years, reaching 85.7% in 2023 and 100% in the first half of 2024.

In a subgroup of 52 patients, penicillin resistance also demonstrated a statistically significant increase (Table 4). During the early years, resistance levels were low to moderate, with 0% in 2020 and 11.8% in 2021. In contrast, resistance rates rose to 69.2% in 2023 and 83.3% in 2024.

Fluconazole resistance, analyzed in a smaller subgroup of nine patients (Table 5), did not show a statistically significant trend, with variable resistance rates across the years.

*Ureaplasma urealyticum* resistance was examined in a group of 44 patients (Table 6). During the first three years (2020–2022), resistance patterns were mixed, with the proportion of MDR cases increasing from 38.5% in 2020 to 63.6% in 2022. However, overall, no statistically significant trend was observed. Notably, no *Ureaplasma* cases were detected during the last two years of the study period (2023–2024).

The annual trend in AMR, defined as the presence of at least one resistance per patient (Table 7), did not show significant variation across the years. Nonetheless, a consistently high prevalence of resistance was observed, with nearly all patients exhibiting some form of antimicrobial resistance in 2023 and 2024.

Trends in resistance to various therapeutic classes, including tetracyclines, quinolones (ciprofloxacin, levofloxacin, or moxifloxacin), nitroimidazoles (metronidazole), lincosamides (clindamycin), and macrolides (clarithromycin), are presented in Table 8.

### 3.1. Resistance Trends by Antimicrobial Class

- Tetracyclines: A substantial proportion of isolates (75.8%, 185 out of 244) were resistant to tetracyclines. This high level of resistance was consistently observed throughout the study period, peaking at 97.4% in both 2023 and the first half of 2024. Resistance rates were lower in 2020 and 2021, at 64.3% and 66.3%, respectively.
- Quinolones: Resistance to quinolones was also common, affecting 68.4% of isolates (167 out of 244). The highest rates were reported in 2023 (97.4%) and in the first half of 2024 (95.7%). Earlier years showed slightly lower resistance levels, with 52.4% in 2020 and 55.8% in 2021.
- Nitroimidazoles: Resistance to nitroimidazoles (metronidazole) was found in 39.3% of patients (96 out of 244). The highest resistance rates occurred in 2023 (60.5%) and the first half of 2024 (56.5%). In comparison, resistance was significantly lower in 2020 (21.4%) and 2021 (33.7%).
- Lincosamides: Resistance to lincosamides (clindamycin) was observed in 50.4% of isolates (123 out of 244). The highest values were again recorded in 2023 (60.5%) and early 2024 (56.5%). Resistance was 42.9% in 2020 and slightly increased to 45.3% in 2021.
- Macrolides: Macrolide resistance (clarithromycin) remained low across the entire cohort, detected in only 2.9% of isolates (7 out of 244), with no significant variation over the years.

### 3.2. Resistance Trends by Year

- In 2020, resistance rates were as follows: tetracyclines 64.3%, quinolones 52.4%, nitroimidazoles 21.4%, lincosamides 42.9%, and macrolides 4.8%.
- In 2021, resistance to tetracyclines was 66.3%, to quinolones 55.8%, to nitroimidazoles 33.7%, to lincosamides 45.3%, and to macrolides 3.2%.
- In 2022, resistance levels increased across most classes: tetracyclines 78.3%, quinolones 71.7%, nitroimidazoles 41.3%, lincosamides 56.5%, and macrolides 4.3%.
- In 2023, resistance peaked for several classes, with rates of 97.4% for both tetracyclines and quinolones, 60.5% for nitroimidazoles, 60.5% for lincosamides, and 0% for macrolides.
- In the first half of 2024, resistance remained high at 95.7% for tetracyclines and quinolones, 56.5% for nitroimidazoles, 56.5% for lincosamides, and 0% for macrolides.

**Figure 1:**
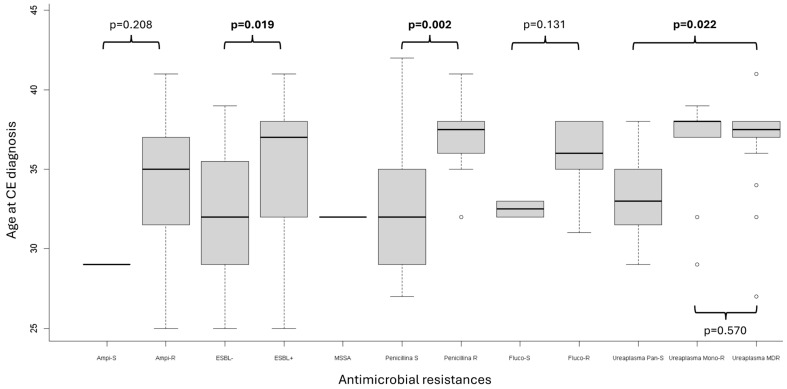
Relationship between the prevalence of AMR and age at CE diagnosis. S = sensitive; R = resistant; Ampi-S = ampicillin-sensitive; Ampi-R = ampicillin resistant; ESBL− = extended-spectrum beta-lactamase negative; ESBL+ = extended-spectrum beta-lactamase positive; MSSA = methicillin-susceptible *Staphylococcus aureus*; Fluco-S = fluconazole-sensitive; Fluco-R = fluconazole-resistant; Pan-S = pan-sensitive; Mono-R = mono-resistant; MDR = multidrug-resistant; *p* = *p*-value; CE = chronic endometritis. Please note that certain subgroups, such as those related to ampicillin and fluconazole resistance, have a small sample size (see text for details).

The association between the prevalence of specific AMR and age at CE diagnosis is illustrated in Figure 1. Both ESBL positivity and penicillin resistance showed a significant increase with advancing age (*p* = 0.019 and *p* = 0.002, respectively). In contrast, no significant association with age was observed for ampicillin resistance, fluconazole resistance, or *Ureaplasma urealyticum* resistance, whether MONO-R or MDR.

## 4. Discussion

This study aimed to determine the prevalence of pathogenic microorganisms and assess AMR trends in endometrial cultures from 244 patients with CE between 2020 and 2024.

Among the pathogens identified, *Enterococcus faecalis* was the most prevalent, accounting for 26.2% of all infections. This finding aligns with previous research [2,3] reporting that *Enterococcus* species are frequently implicated in CE, likely due to translocation from the intestinal tract to the genitourinary system. *Escherichia coli* was the second most common pathogen (19.3%), followed by *Klebsiella pneumoniae* and *Ureaplasma urealyticum*, confirming the microbial heterogeneity associated with CE.

Importantly, our findings demonstrate a significant increase in AMR among CE-associated microorganisms over the study period. This trend suggests that conventional first-line antimicrobials may be losing effectiveness in a growing proportion of patients.

In a subgroup of 65 patients, 98.5% showed resistance to ampicillin, with no significant year-to-year variation. In contrast, penicillin resistance rose markedly from 0% in 2020 to 69.2% in 2023 and 83.3% in 2024. Similarly, the prevalence of ESBL+ isolates increased significantly, from 0% in 2020 to 85.7% in 2023 and 100% in early 2024. This sharp rise reflects a dramatic reduction in the efficacy of beta-lactam antibiotics, which are widely used to treat bacterial infections. One major concern is the emergence of carbapenemase-producing Enterobacteriaceae (CPE) from ESBL strains [18], which often leaves no oral therapeutic alternatives and necessitates intravenous therapy [19]. This increases healthcare costs and the risk of MDR strain transmission. Therefore, robust antimicrobial stewardship programs are urgently needed to control the spread of ESBL-producing organisms.

The management of CE may be further challenged by high rates of resistance to tetracyclines (75.8%), quinolones (68.4%), and nitroimidazoles (39.3%) observed in our cohort.

Doxycycline, a tetracycline derivative, has recently attracted renewed interest as a post-exposure prophylactic agent for sexually transmitted infections (doxy-PEP). However, preliminary in vitro studies suggest that doxy-PEP exposure may induce increased doxycycline resistance [20]. Furthermore, mutations linked to doxycycline resistance have been hypothesized to co-occur with mutations conferring resistance to colistin [21], raising concerns that misuse of one antibiotic could promote resistance across other classes. Although doxycycline is cost-effective, its widespread use may contribute to increased selective pressure and the proliferation of MDR bacteria [22]. This may lead to a further escalation of tetracycline resistance in the future.

Quinolone resistance is already a recognized problem [23]. According to the EARS-Net 2023 surveillance report, *Escherichia coli* quinolone resistance in Europe averages around 30%, and was 31.6% in Italy in 2022. For *Klebsiella pneumoniae*, the resistance rate is even higher at 48.7%. These figures are more favorable than the 68.4% observed in our cohort, even considering that AMR in Southern Italy is typically worse than the national average (source: ECDC report).

Although lower than for other antimicrobials, nitroimidazole resistance (39.3%) also appears to be increasing. It is important to note that metronidazole, the most widely used nitroimidazole, has a relatively narrow antimicrobial spectrum, targeting primarily anaerobic bacteria. This limits its clinical utility in treating CE. Nevertheless, it remains in occasional empirical use in some clinical settings, which justifies its inclusion in our analysis for descriptive purposes.

The low prevalence of macrolide resistance (2.9%) suggests that these agents may still retain efficacy in selected cases. However, their use should remain cautious to prevent resistance development. The widespread use of azithromycin during the COVID-19 pandemic has already contributed to increased macrolide resistance in group B streptococci [24]. For this reason, our findings may also deteriorate in the near future.

Another notable finding of our study is the association between patient age and the prevalence of certain AMRs. While no significant age-related differences were observed for resistance to ampicillin, fluconazole, or *Ureaplasma urealyticum* (both MONO-R and MDR strains), a significant correlation emerged between increasing age and the prevalence of ESBL-producing organisms and penicillin-resistant isolates. The elevated resistance rates in older patients may reflect age-related immunological changes or cumulative antimicrobial exposure over time [25]. These observations highlight the importance of timely diagnosis and treatment in younger patients to mitigate future resistance risks.

Taken together, our results emphasize the urgent need for improved diagnostic and therapeutic strategies in the management of CE. Delayed or ineffective treatment may lead to reproductive complications, such as failure to conceive spontaneously or unsuccessful embryo implantation following IVF [4], with potential psychological consequences for affected individuals and couples [26]. Additionally, treatment failures contribute to increased healthcare expenditures due to repeated antimicrobial courses and additional IVF cycles.

The high prevalence of AMR observed in this study raises concerns about the widespread use of empirical antimicrobial regimens in CE management. Nonetheless, several limitations must be acknowledged. First, the data were derived from a single geographic area (Apulia region, Southern Italy) and collected over a relatively short time period, which included phases of reduced access to care during the COVID-19 pandemic. Second, the small sample size in certain subgroups, particularly those analyzed for fluconazole and penicillin resistance, limits statistical power and increases the uncertainty of those estimates. As such, these results should be considered exploratory and interpreted with caution. Future research on larger and more diverse populations is warranted to validate and expand upon these findings. Until these data are available, a targeted diagnostic approach based on hysteroscopy, endometrial biopsy, microbial culture, and susceptibility testing remains the most rational strategy for guiding CE treatment.

In conclusion, the increasing AMR among CE-associated pathogens underscores the need for a comprehensive response [27]. Priorities include the standardization of diagnostic protocols to enable tailored therapies, enhanced surveillance systems to track evolving resistance patterns, implementation of antimicrobial stewardship programs to optimize prescribing practices [28], and improved patient education on the dangers of self-medication [29]. Containing AMR is especially critical in CE given its potential to compromise female fertility.

## 5. Conclusions

The findings of this study highlight the growing concern regarding AMR in CE, with important implications for both clinical management and public health. The high rates of resistance to commonly used antimicrobials underscore the urgent need for more accurate targeted diagnostic and therapeutic strategies. Strengthening surveillance systems, promoting antimicrobial stewardship programs, and improving patient education may contribute to mitigating the spread of AMR in this context.

## Figures and Tables

**Table 1:** Description of pathogens isolated by year in the 244 enrolled patients.

Germ Isolated	Total (N = 244)	2020 (N = 42)	2021 (N = 95)	2022 (N = 46)	2023 (N = 38)	2024 * (N = 23)
*Candida albicans*	3 (1.2%)	1 (2.4%)	1 (1.1%)	1 (2.2%)	0 (0%)	0 (0%)
*Candida glabrata*	1 (0.4%)	0 (0%)	1 (1.1%)	0 (0%)	0 (0%)	0 (0%)
*Candida krusei*	1 (0.4%)	0 (0%)	1 (1.1%)	0 (0%)	0 (0%)	0 (0%)
*Candida Krusei*	1 (0.4%)	0 (0%)	0 (0%)	0 (0%)	1 (2.6%)	0 (0%)
*Candida parapsilosis*	3 (1.2%)	1 (2.4%)	1 (1.1%)	0 (0%)	0 (0%)	1 (4.3%)
*Enterococcus faecalis*	64 (26.2%)	7 (16.7%)	25 (26.3%)	15 (32.6%)	10 (26.3%)	7 (30.4%)
*Escherichia coli*	47 (19.3%)	6 (14.3%)	18 (18.9%)	9 (19.6%)	7 (18.4%)	7 (30.4%)
*Klebsiella pnuemoniae*	24 (9.8%)	5 (11.9%)	8 (8.4%)	2 (4.3%)	7 (18.4%)	2 (8.7%)
*Mycoplasma hominis*	4 (1.6%)	1 (2.4%)	2 (2.1%)	1 (2.2%)	0 (0%)	0 (0%)
*Proteus mirabilis*	1 (0.4%)	0 (0%)	1 (1.1%)	0 (0%)	0 (0%)	0 (0%)
*Staphylococcus saprophyticus*	1 (0.4%)	0 (0%)	0 (0%)	0 (0%)	1 (2.6%)	0 (0%)
*Staphylococcus aureus*	2 (0.8%)	0 (0%)	1 (1.1%)	1 (2.2%)	0 (0%)	0 (0%)
*Staphylococcus haemolyticus*	1 (0.4%)	0 (0%)	1 (1.1%)	0 (0%)	0 (0%)	0 (0%)
*Streptococcus agalactieae* (*Group B Streptococcus*)	34 (13.9%)	7 (16.7%)	10 (10.5%)	7 (15.2%)	6 (15.8%)	4 (17.4%)
*Streptococcus bovis*	17 (7.0%)	2 (4.8%)	7 (7.4%)	0 (0%)	6 (15.8%)	2 (8.7%)
*Ureaplasma urealiticum*	40 (16.4%)	12 (28.6%)	18 (18.9%)	10 (21.7%)	0 (0%)	0 (0%)

* Data for 2024 cover only the first 6 months. In both cases involving Staphylococcus aureus, the strains were identified as methicillin-sensitive (MSSA).

**Table 2:** Ampicillin resistance trends.

Years of Diagnosis	Total (N = 65)	Amp-S (N = 1)	Amp-R (N = 64)	*p*-Value
2020	7 (10.8%)	0 (0%)	7 (100%)	0.517
2021	26 (40.0%)	1 (3.8%)	25 (96.2%)	
2022	15 (23.1%)	0 (0%)	15 (100%)	
2023	10 (15.4%)	0 (0%)	10 (100%)	
2024 *	7 (10.8%)	0 (0%)	7 (100%)	

* Data for 2024 cover only the first 6 months. Amp-S = ampicillin-sensitive; Amp-R = ampicillin-resistant.

**Table 3:** Trends in ESBL positivity.

Years of Diagnosis	Total (N = 72)	ESBL− (N = 47)	ESBL+ (N = 25)	*p*-Value
2020	11 (15.3%)	11 (100%)	0 (0%)	**<0.001**
2021	27 (37.5%)	24 (88.9%)	3 (11.1%)	
2022	11 (15.3%)	10 (90.9%)	1 (9.1%)	
2023	14 (19.4%)	2 (14.3%)	12 (85.7%)	
2024 *	9 (12.5%)	0 (0%)	9 (100%)	

* Data for 2024 cover only the first 6 months. ESLB+: germs exhibiting resistance to both penicillins and cephalosporin. Bold indicates statistical significance (*p* < 0.05).

**Table 4:** Penicillin resistance trends.

Years of Diagnosis	Total (N = 52)	Pen-S (N = 36)	Pen-R (N = 16)	*p*-Value
2020	9 (17.3%)	9 (100%)	0 (0%)	**<0.001**
2021	17 (32.7%)	15 (88.2%)	2 (11.8%)	
2022	7 (13.5%)	7 (100%)	0 (0%)	
2023	13 (25.0%)	4 (30.8%)	9 (69.2%)	
2024 *	6 (11.5%)	1 (16.7%)	5 (83.3%)	

* Data for 2024 cover only the first 6 months. Pen-S = penicillin-sensitive, Pen-R = penicillin-resistant. Bold indicates statistical significance (*p* < 0.05).

**Table 5:** Fluconazole resistance trends.

Years of Diagnosis	Total (N = 9)	Fluco-S (N = 4)	Fluco-R (N = 5)	*p*-Value
2020	2 (22.2%)	0 (0%)	2 (100%)	0.264
2021	4 (44.4%)	2 (50.0%)	2 (50.0%)	
2022	1 (11.1%)	1 (100%)	0 (0%)	
2023	1 (11.1%)	0 (0%)	1 (100%)	
2024 *	1 (11.1%)	1 (100%)	0 (0%)	

* Data for 2024 cover only the first 6 months. Fluco-S = fluconazole-sensitive, Fluco-R = fluconazole-resistant.

**Table 6:** *Ureaplasma urealyticum* resistance trend.

Years of Diagnosis	Total (N = 44)	Ureaplasma Pan-S (N = 7)	Ureaplasma MONO-R (N = 17)	Ureaplasma MDR (N = 20)	*p*-Value
2020	13 (29.5%)	2 (15.6%)	6 (46.2%)	5 (38.5%)	0.317
2021	20 (45.5%)	4 (20.0%)	8 (40.0%)	8 (40.0%)	
2022	11 (25.0%)	1 (9.1%)	3 (27.3%)	7 (63.6%)	
2023	0 (0%)	-	-	-	
2024 *	0 (0%)	-	-	-	

* Data for 2024 cover only the first 6 months. Pan-S = sensitive to all antibiotics tested; MONO-R monodrug-resistant; MDR = multidrug-resistant.

**Table 7:** Overall resistance trends.

Years of Diagnosis	Total (N = 244)	No Resistance Detected (N = 16)	At Least One Resistance Detected (N = 228)	*p*-Value
2020	42 (17.2%)	2 (4.8%)	40 (95.2%)	0.218
2021	95 (38.9%)	10 (10.5%)	85 (89.5%)	
2022	46 (18.9%)	3 (6.5%)	43 (93.5%)	
2023	38 (15.6%)	0 (0%)	38 (100%)	
2024 *	23 (9.4%)	1 (4.3%)	22 (95.7%)	

* Data for 2024 cover only the first 6 months.

**Table 8:** Trends in resistance to therapeutic classes.

	Tetracycline Resistance	Quinolone Resistance	Nitroimidazole Resistance	Lincosamide Resistance	Macrolide Resistance
Total (N = 244)					
Yes	185 (75.8%)	167 (68.4%)	96 (39.3%)	123 (50.4%)	7 (2.9%)
No	50 (20.5%)	68 (27.9%)	23 (9.4%)	40 (16.4%)	37 (15.2%)
NA	9 (3.7%)	9 (3.7%)	125 (51.2%)	81 (33.2%)	200 (82.0%)
2020 (N = 42)					
Yes	27 (64.3%)	22 (52.4%)	9 (21.4%)	18 (42.9%)	2 (4.8%)
No	13 (31.0%)	18 (42.9%)	7 (16.7%)	11 (26.2%)	11 (26.2%)
NA	2 (4.8%)	2 (4.8%)	26 (61.9%)	13 (31.0%)	29 (69.0%)
2021 (N = 95)					
Yes	63 (66.3%)	53 (55.8%)	32 (33.7%)	43 (45.3%)	3 (3.2%)
No	28 (29.5%)	38 (40.0%)	12 (12.6%)	21 (22.1%)	17 (17.9%
NA	4 (4.2%)	4 (4.2%)	51 (53.7%)	31 (32.6%)	75 (78.9%)
2022 (N = 46)					
Yes	36 (78.3%)	33 (71.7%)	19 (41.3%)	26 (56.5%)	2 (4.3%)
No	9 (19.6%)	12 (26.1%)	4 (8.7%)	8 (17.4%)	9 (19.6%)
NA	1 (2.2%)	1 (2.2%)	23 (50.0%)	12 (26.1%)	35 (76.1%)
2023 (N = 38)					
Yes	37 (97.4%)	37 (97.4%)	23 (60.5%)	23 (60.5%)	0 (0%)
No	0 (0%)	0 (0%)	0 (0%)	0 (0%)	0 (0%)
NA	1 (2.6%)	1 (2.6%)	15 (39.5%)	15 (39.5%)	38 (100%)
2024 * (N = 23)					
Yes	22 (95.7%)	22 (95.7%)	13 (56.5%)	13 (56.5%)	0 (0%)
No	0 (0%)	0 (0%)	0 (0%)	0 (0%)	0 (0%)
NA	1 (4.3%)	1 (4.3%)	10 (43.5%)	10 (43.5%)	23 (100%)

* Data for 2024 cover only the first 6 months. NA = not applicable.

## Data Availability

The data that support the findings of this study are not publicly available due to privacy reasons but are available from Pierpaolo Nicolì upon reasonable request.
